# Altered metabolic and inflammatory transcriptomics after cardiac surgery in neonates with congenital heart disease

**DOI:** 10.1038/s41598-021-83882-x

**Published:** 2021-03-02

**Authors:** Parag N. Jain, Matthew Robertson, Javier J. Lasa, Lara Shekerdemian, Danielle Guffey, Yuhao Zhang, Krithika Lingappan, Paul Checchia, Cristian Coarfa

**Affiliations:** 1 Baylor College of Medicine and Texas Children’s Hospital, Houston, TX USA; 2 Baylor College of Medicine, Houston, TX USA

**Keywords:** Transcriptomics, Cardiovascular genetics, Congenital heart defects, Heart failure

## Abstract

The study examines the whole blood transcriptome profile before and after cardiopulmonary bypass (CPB) in neonates with hypoplastic left heart syndrome (HLHS), a severe form of congenital heart disease, that can develop low cardiac output syndrome (LCOS). Whole blood mRNA transcriptome profiles of 13 neonates with HLHS before and after their first palliative surgery were analyzed to determine differentially expressed genes and pathways. The median age and weight at surgery were 4 days and 3.2 kg, respectively. Of the 13 patients, 8 developed LCOS. There was no significant difference between CPB, aortic cross clamp, deep hypothermic cardiac arrest times between patients that develop LCOS and those that do not. Upon comparing differential gene expression profiles between patients that develop LCOS and those that do not in pre-operative samples, 1 gene was up-regulated and 13 were down regulated. In the post-operative samples, 4 genes were up-regulated, and 4 genes were down regulated when patients that develop LCOS were compared to those that do not. When comparing post-operative samples to pre-operative samples in the patients that do not develop LCOS, 1484 genes were up-regulated, and 1388 genes were down regulated; while patients that developed LCOS had 2423 up-regulated genes, and 2414 down regulated genes for the same pre to post-operative comparison. Pathway analysis revealed differential regulation of inflammatory pathways (IL signaling, PDGF, NOTCH1, NGF, GPCR) and metabolic pathways (heme metabolism, oxidative phosphorylation, protein metabolism including amino acid and derivatives, fatty acid metabolism, TCA cycle and respiratory electron transport chain). By identifying altered transcriptome profiles related to inflammation and metabolism in neonates with HLHS who develop LCOS after CPB, this study opens for exploration novel pathways and potential therapeutic targets to improve outcomes in this high-risk population.

## Introduction

Congenital heart defects are the most common birth defects, affecting nearly 40,000 infants each year in the United States of America^1–3^. The majority of forms of congenital heart disease (CHD) presenting during the neonatal period require surgery on cardiopulmonary bypass (CPB) in early infancy and multiple subsequent surgeries. Of these, hypoplastic left heart syndrome (HLHS) remains the most critical form of congenital heart disease, requiring as a first stage of surgical palliation the Norwood operation or its modifications, typically performed during the early neonatal period. The Norwood operation is one of the highest risk operations for CHD, with mortality rate as high as 19%^4^.

Cardiopulmonary bypass (CPB) activates an inflammatory response that closely resembles the systemic inflammatory response syndrome (SIRS), which is characterized by alterations in cardiovascular and pulmonary function, fever, fluid retention, coagulopathy, and multisystem organ dysfunction/failure (MODS)^5,6^. The degree of morbidity attributed to the post-CPB inflammatory response is correlated to its severity^7^. In particular, low cardiac output syndrome (LCOS) has been associated with this inflammatory response which contributes to post-operative morbidity and mortality^8,9^.

The cellular and molecular mechanisms regulating the systemic inflammatory response to CPB are not completely understood, but are thought to include complement activation, cytokine release, and endothelial cell activation, leading to myocardial and vascular injury^10–12^. Differences in the host’s genotype may determine their individual pro or anti-inflammatory response profile, and thus the magnitude of their systemic inflammatory response to CPB. Whole blood transcriptomic studies have accounted for the heterogenous inflammatory response seen after CPB in adults^13–16^. However, there are no studies exploring whole blood transcriptome profile in neonates with critical CHD, and in particular HLHS. In this study we hypothesized that children with HLHS will have unique transcriptome profiles before and after their Norwood operation, and that they would correlate with the development of a post-operative LCOS.

## Methods

### Patient enrollment

This was a single-institution prospective observational study performed at the Texas Children’s Hospital Heart Center. The study was approved by the Institutional Review Board (IRB) of Baylor College of Medicine. All experiments were performed according to the guidelines approved by IRB. Since the subjects were minor, an informed consent was obtained from legal guardian/parent of each enrolled patients. We enrolled 13 neonates diagnosed with HLHS, between July 2016 and August 2017, who were scheduled for a Norwood procedure. Exclusion criteria involved patients with weight < 3 kg at the time of surgery, a known prenatal chromosomal disorder, or gestational age < 34 weeks.

### Anesthetic and surgical technique

Perioperative procedures and techniques (including anesthesia and surgery) were standardized per institution protocol and have been reported previously by Andropoulos et al.^17^. Briefly, the patient was anesthetized using isoflurane, fentanyl and midazolam. All patients received one dose of IV methylprednisolone (30 mg/kg/dose) on induction of anesthesia. None of the patients received additional blood transfusion before obtaining second sample after surgery.

Bypass flow rates of 150 cc/kg/min were utilized, and pH–stat blood gas management was used for all cases. For aortic arch reconstruction, regional cerebral perfusion (RCP) was utilized at 18 °C, along with deep hypothermic cardiac arrest for brief periods, and never for more than 20 min. Hematocrit was maintained at 30–35% during cooling and hypothermic periods and increased to 40–45% during rewarming. Regional oxygen saturations of the brain (rSO_2_) were monitored throughout the perioperative period with a protocol that attempted to maintain rSO_2_ > 50% before and after bypass and > 90% while on bypass (INVOS 5100B; Somanetics, Inc., Troy, MI, USA).

### Blood samples

Initial blood samples were collected from an existing arterial or venous catheter in all patients before induction of anesthesia and then again, 24 h post-operatively.

### RNA purification

Blood samples were collected in PAXgene Blood RNA Tubes (Qiagen, Germantown, CA, USA). RNA was extracted using Qiagen PAXgene Blood miRNA kit (Qiagen, Germantown, CA, USA) using automated protocol.

### RNA sequencing protocol

Total RNA quality assessment was done using BioRad Experion Automated Electrophoresis Station (Biorad, Hercules, CA, USA) and quantified using Qubit 3.0 fluorometer (Qubit RNA BR Assay Kit) (Life Technologies Corporation, Carlsbad, CA, USA). Sample libraries were prepared using the Illumina TruSeq Stranded mRNA Sample Preparation Kit (Cat # 20020594) and TruSeq RNA Single Index Set A Index (Cat # 20020492) (Illumina Inc., San Diego, CA, USA). Two rounds of purification were performed and during the second elution the RNA was fragmented and primed for cDNA synthesis. DNA was subjected to end repair, A-tailing and adapter ligation. Post ligation cleanup was performed using AMpure XP beads (Agencourt, Beverly, MA, USA). DNA fragments were amplified using PCR and purified using AMpure XP beads (Agencourt, Beverly, MA, USA). The quality of the amplified libraries was checked on an Agilent 4200 Tape Station System (Agilent Technologies, Santa Clara, CA, USA) using the D1000 Screen Tape Assay. The libraries were standardized to 2 nM, equal volumes of these libraries were pooled, and then the pooled library was sequenced using 100 bp paired-end reads on the Illumina HiSeq 2000 platform (Illumina Inc., San Diego, CA, USA). Samples were demultiplexed using Casava (v1.8, Illumina)^18^.

### RNA-seq alignment and quantification

Fastq data files were trimmed using Trim Galore (version 0.5.0)^19^. The trimmed sequences were aligned against the human genome (GRCH38) using HISAT2 before sorting reads using samtools^20,21^. Finally, reads were quantified using featureCounts (version 1.6.4) against the GENCODE gene reference^22,23^.

### Differential expression analysis

The counts matrix generated using featureCounts was processed in the R statistical software environment (version 3.5) to determine differentially expressed genes for the reported comparisons. The R package RUVseq was used to remove unwanted variation using the RUVr method^24^. DESEQ2 was then used to determine differential gene expression using the likelihood ratio test^25^. Significance was achieved for fdr-adjusted *p*-value < 0.05 and fold change exceeding 1.5×.

### Principal component analysis and data visualization

Data was visualized using GraphPad Prism version 8 unless indicated differently. PCA, heatmaps and volcano plots were generated using the R statistical software environment. Circos plots were generated using the Circos software (version 0.69) and Circos Table Viewer software (version 0.63)^26^.

### Gene set enrichment analysis (GSEA)

All the genes detected for a comparison were ranked according to their log2 fold change in expression regardless of statistical significance. GSEA was performed using the Java-based GSEA software (version 3.0) on this ranked gene lists against the entire MSigDB database (version 6.1)^27,28^. with 1000 permutations; significance was achieved for a *q*-value less than or equal to 0.25, per the GSEA developers recommended practices.

### Quantitative real time PCR analysis (qPCR)

Quantitative PCR was performed using the QuantStudio 7 Flex real-time PCR detection system (ThermoFIsher) and SYBR Green (#1725274, Bio-Red). The thermal cycling conditions used were as follows: one cycle at 95 °C for 1 min, 40 cycles at 95 °C for 15 s, and 60 °C for 15 s. The primers used in real-time PCR test were listed as follows: GRB2 forward primer AATTATGTCACCCCCGTGAA, GRB2 reverse primer TGTTCTGCACTCCCTCACAG; LYN forward primer ACCAAGGTGGCTGTGAAAAC, LYN reverse primer CTGGTGACCACAGCGTAGAG; PRKACB forward primer CTTTGGGTTTGCCAAAAGAG, PRKACB reverse primer CTAATGCCCACCAATCCACT; PIK3CB forward primer GCACATTCCTGCTGTCTCAG, PIK3CB reverse primer TCACGGCATTCAGTTTGATT; PIK3R3 forward primer AGCCTGTGGAAATGGCATAG, PIK3R3 reverse primer CTCTCATGAAGGAGGCCAAG; STAT5B forward primer GTCCCAGAAACACCTCCAGA, STAT5B reverse primer TCAGGCTCTCCTGGTACTGG; PTPN6 forward primer TGCTTATGGGCCCTACTCTG, PTPN6 reverse primer TAATGCCAGATCTCCCGAAT; AKT3 forward primer ACGACCAAAGCCAAACACAT, AKT3 reverse primer TCTTGCCTCTGCAGTCTGTC; IL2RB forward primer AGAAGTGCTGGAGAGGGACA, IL2RB reverse primer CGGGAGGTGGAAGAAGAAGT; CSF2RA forward primer GAATGTTCGTGCACATTTCG, CSF2RA reverse primer ACCCTCCCTTCCTGAATTTG; IL5RA forward primer CTCTGCTATCAGGCCCTTTG, IL5RA reverse primer AGCAATGGATTGGAAAAGCA; IL7R forward primer GGTTTGCCTAGTGCTTTTGC, IL7R reverse primer GGCAGAATGCCATCCTTTTA; MAPK3 forward primer GGGAGGGGAGGAGTGGAG, MAPK3 reverse primer GCTGCCCCTTCACCATCT; CYC1 forward primer CACGGAGGATGAAGCTAAGG, CYC1 reverse primer AGCCTCACTGTTGGGGTATG; COX8A forward primer AGCTTGGGATCATGGAATTG, COX8A reverse primer AGAACGGACCCCTTCACTCT; UQCRC1 forward primer ACTGTTAGACCTCGCCCAGA, UQCRC1 reverse primer GGGCAAAAGGTAGAGCATCA; LDHA forward primer GTACTGCATTTGCCCCTTGA, LDHA reverse primer CTGGATCCCAGGATGTGACT; GYPA forward primer GAACTGTGTCGGAGCACTCA, GYPA reverse primer ATGTCCGGTTTGCACATCTT; ALAS2 forward primer TTCCTACTTCGGGAACATGG, ALAS2 reverse primer GAGGCACACAACAAAGCAGA; FOXO3 forward primer GGCGGACTTTGTGTTTGTTT, FOXO3 reverse primer AAGCCACCTGAAATCACACC; β-ACTIN forward primer CATCGAGCACGGCATCGTCA, β-ACTIN reverse primer TAGCACAGCCTGGATAGCAAC. Relative mRNA levels were calculated using the 2^−ΔΔCT^ method and normalized by β-actin in the same sample.

### Whole blood deconvolution

The gene expression read counts profiles were filtered to remove poorly expressed genes, then upper quartile normalized was applied, and finally the counts per million (CPM) were determined for each gene using the R package EdgeR^29^. The relative cell abundance of 22 different immune cells was determined based on the CPM transcriptomic profile for all the samples using the LM22 signature and CIBERSORTx method^30^.

### Clinical data analysis

Our primary clinical outcome data was development of low cardiac output syndrome (LCOS) as defined by at least one of the following during the first 48 h period: VIS > 15 at any time, addition of a new vasoactive agent for patients already on inotropic or vasopressor support or a new initiation of vasoactive support (inotropes or vasopressors or milrinone) after a 24 h period with no support.

### Statistical analysis

Patient characteristics and outcomes were compared by LCOS using t-test, Wilcoxon ranksum test, Chi-square test and Fisher's exact test using Stata v 15. Statistical analysis of the quantitative real time PCR data was perform using the python library scipy.

## Results

### Patient characteristics (Table 1)

The median age of patients at surgery was 4 days (IQR 2–6 days) with median gestational age of 39 weeks and median weight at surgery of 3.2 kg. The patients were all Caucasian, predominantly male and of non-Hispanic ethnicity. We stratified the patients by the primary clinical outcome—one group that developed LCOS and the other that did not develop LCOS (No-LCOS). Of the total population, eight patients developed LCOS. Patients that developed LCOS were more likely to have a lower birth weight (*p* < 0.05) (Table 1). There was no association between the development of LCOS and gestational age, age at time of surgery, CPB time, aortic cross clamp time, deep hypothermic cardiac arrest, or length of stay in the hospital and intensive care unit.

**Table 1:** Demographic and clinical patient characteristics.

Variable	No LCOS (n = 5) Median (IQR)	LCOS (n = 8) Median (IQR)	*p *Value
Male	4	8	0.385
Race–Caucasian	5	8	1.00
Gestational age (weeks)			0.804
34	0	1	
35	0	1	
36	1	0	
39	4	6	
Birth weight (kg)	3.5 (3.4–3.7)	3 (2.8–3.2)	0.028
CPB (min)	184 (178–210)	211 (163–230)	0.883
Aortic cross clamp (min)	96 (93–124)	99.5 (82.5–117.5)	0.341
Circulatory arrest (min)	8 (7–11)	8 (7–14.5)	0.765
ACP (min)	85 (77–86)	76 (61.5–93.5)	0.77
Hospital LOS (days)	47 (46–140)	56 (42–132)	0.77
CICU LOS (days)	29.6 (24.8–38.7)	32.8 (19.7–56.1)	1.00
Mechanical ventilation duration (days)	3 (1.8–4.3)	4 (2.8–10.9)	0.223

*LCOS* Low cardiac output syndrome, *CPB* Cardiopulmonary bypass, *ACP* Antegrade cerebral perfusion, *LOS* Length of stay.

The median mechanical ventilation duration was 3.81 days, median ICU and hospital length of stay was 30 and 55 days respectively. There were 2 hospital deaths. There was no difference between the groups with respect to rate of post-operative complications (Table 2).

**Table 2:** Post-operative complications.

Variable	No LCOS (n = 5)	LCOS (n = 8)	*p* Value
Arrythmia requiring therapy (n)	2	2	1.00
Post-op ECMO (n)	0	1	1.00
Sepsis (n)	0	1	1.00
CPR prior to surgery (n)	0	0	1.00
Pre-op infection (n)	0	0	1.00
Left OR with open chest (n)	0	2	0.487

*CPR* Cardiopulmonary resuscitation, *ECMO* Extra corporeal membrane oxygenation, *OR* Operating room.

### Differential gene expression

**Figure 1:**
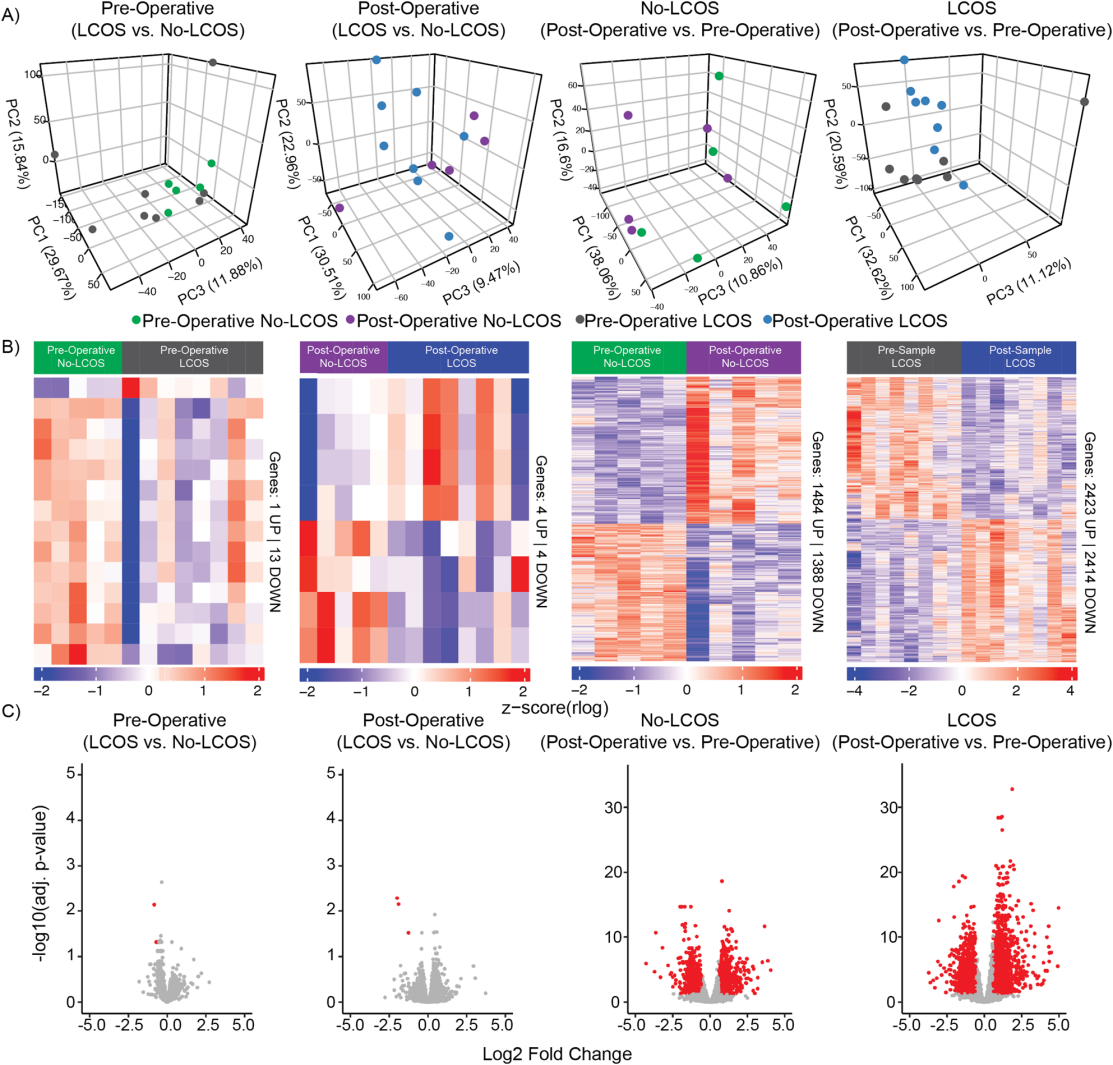
(**A**) Principal Component Analysis (PCA) of the patient transcriptome profiles in different comparisons. (**B**) Heatmap of differentially regulated genes (*q*-value < 0.05, fold change exceeding 1.5×). (**C**) Volcano plots of the different comparisons performed on the HLHS cohort samples where red dots indicate genes with a *q*-value < 0.05 and a linear fold change of at least 1.5×, and grey dots are genes that do not meet these criteria. (R statistical software environment version 3.5 https://www.r-project.org/).

Principal component analysis was performed to reduce the dimensionality of the data and determine if the predominant gene expression variation in the samples could be associated with pre-operative or post-operative status, or with the LCOS outcome. There is an emergent pattern showing that LCOS status can be attributed to the variation observed in the samples after surgery between patients that develop LCOS and those that do not (post-operative LCOS vs. No-LCOS). There is also a pattern present in the patients that develop LCOS when samples before and after surgery are compared (LCOS post-operative vs. pre-operative). However, within the first three principal components there is not a clear delineation between pre-operative and post-operative patients that do not develop LCOS (Fig. 1A).

We considered four different comparisons within the patient cohort. We first examined differential gene expression pre-operatively comparing the LCOS (patients that will develop LCOS) and No-LCOS (patients that will not develop LCOS) groups; then we examined differential gene expression post-operatively comparing LCOS and No-LCOS patient groups. Next, we assessed the transcriptomic impact of the surgical procedure. For the LCOS group we compared post-operative gene expression to pre-operative gene expression, and we did a similar before and after comparison for the No-LCOS group.

There were few genes that were statistically significant when pre-operative patients that do not develop LCOS were compared to patients that develop LCOS. A similar trend was observed in post-operative comparison between LCOS and No-LCOS groups (Supplemental Table 1). In the pre-operative samples (LCOS vs. No-LCOS), 1 gene was up-regulated and 13 were down regulated (*q*-value < 0.05). In the post-operative samples (LCOS vs No-LCOS), 4 genes were up-regulated, and 4 genes were down regulated (*q*-value < 0.05). The surgical intervention had a greater effect on gene expression (Supplemental Table 2). In the No-LCOS group, 1484 genes were up-regulated, and 1388 genes were down regulated (*q*-value < 0.05), while patients that developed LCOS had 2423 up-regulated genes, and 2414 down regulated genes (*q*-value < 0.05) after surgery (Fig. 1B).

**Figure 2:**
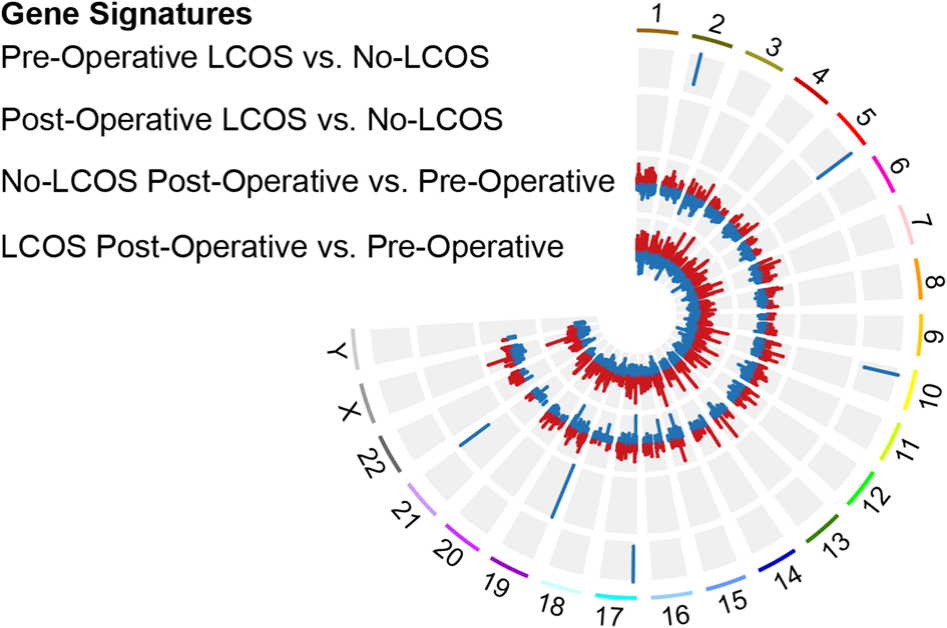
Distribution of differentially regulated genes across the human genome for each comparison performed on the HLHS cohort. The outermost circle represents minimally differential gene regulation in pre-operative samples for patients with and without low cardiac output syndrome (LCOS). The second inner circle compares the differentially regulated genes in post-operative samples of patients with and without LCOS, again showing minimal differential in gene regulation. The third inner circle represents gene expression between pre-operative and post-operative samples in patients who did not develop LCOS, and shows a robust signature. The innermost circle represents the most abundant differential expression, both in terms of number of genes and absolute fold changes, when comparing post-operative samples over pre-operative samples in patients who developed LCOS. Red color indicates increased expression and the blue color indicates decreased expression for the respective comparison. (GraphPad Prism version 8 https://www.graphpad.com/scientific-software/prism/).

Signatures for each comparison were generated by filtering the results for statistical significance (*q*-value < 0.05) and a linear fold change of at least 1.5×. Volcano plots and a Circos plot for these signatures illustrate that patients that develop LCOS have an overall greater fold change in gene expression pre-operative versus post-operative (Figs. 1C and 2); concordantly, the LCOS group shows stronger significance of the gene differences pre-operatively and post-operatively and a larger number of significantly changed genes. In addition, these signatures show no preferential distribution across the human genome (Fig. 2). The deconvolution of the whole blood patient transcriptome and quantification of the relative abundances for 22 different immune cell types was performed to determine if the differences in gene expression could be attributed to differences in the proportion of immune cell types. No significant differences were detected (Supplemental Fig. 1).

### Pathway analysis

**Figure 3:**
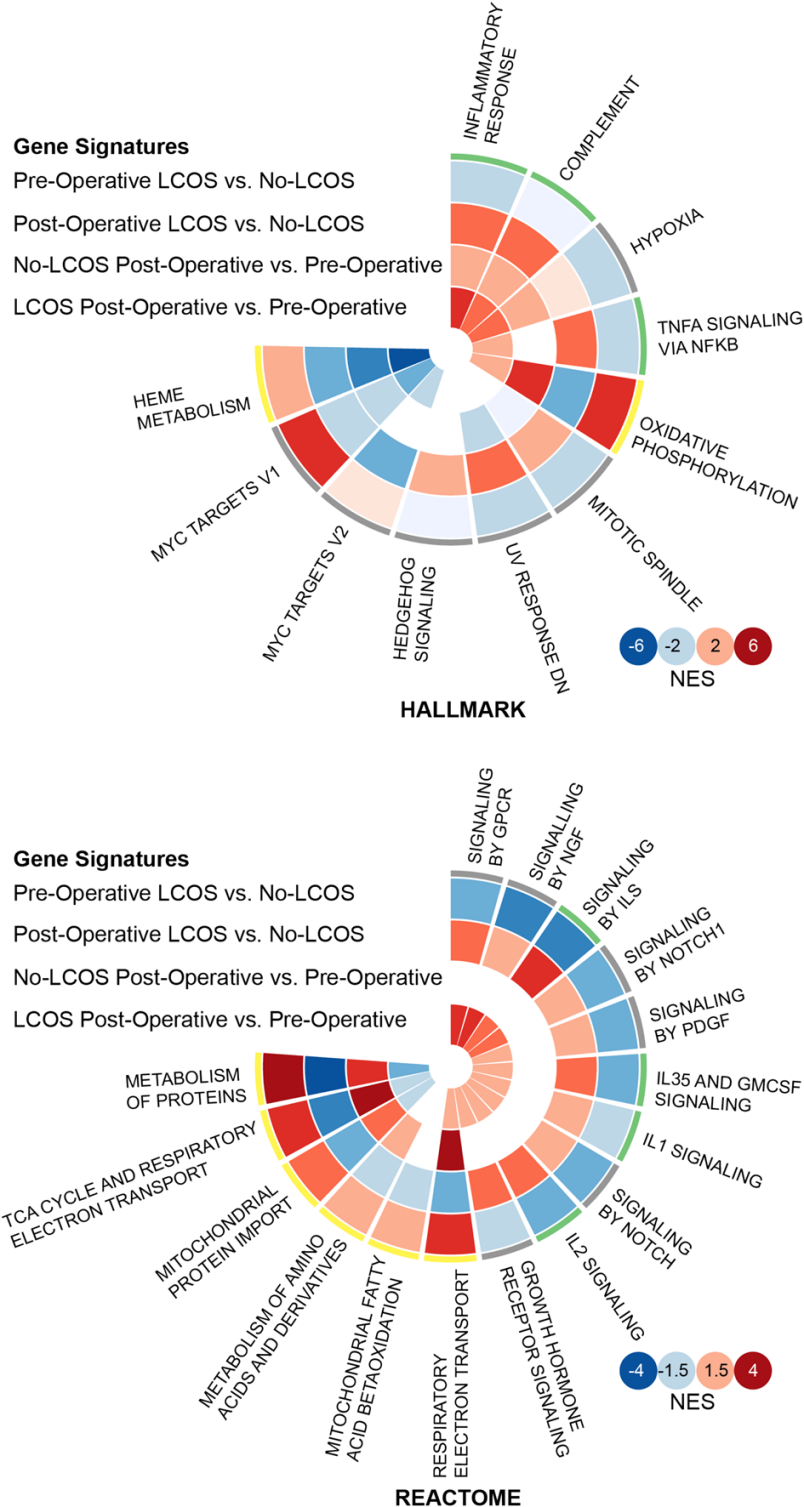
HALLMARK an REACTOME enriched pathways: distribution of Normalized Enrichment Scores (NES) for various pathophysiological pathways. From outside of circle to the inside: outermost circle compares NES scores in Pre-Operative samples of patients who developed LCOS versus No-LCOS, the second circle compares NES scores in Post-Operative samples of patients who developed LCOS vs patients who did not, the third circle compares NES scores between Pre and Post-Operative samples of patients who did not develop LCOS, the innermost circle compares NES scores between Post-Operative and Pre-Operative samples of patients who developed LCOS. (GSEAsoftware version 3.0. https://software.broadinstitute.org/cancer/software/gsea/wiki/index.php/GSEA_v3.0_Release_Notes).

Gene Set Enrichment Analysis (GSEA) was performed on each of the four different comparisons in order to gain insight into the pathways and mechanisms which may regulate LCOS. When comparing pre-operative samples between patients who developed LCOS versus patients who did not (No-LCOS), there was a down regulation of genes controlling inflammatory pathways (IL signaling, PDGF, NOTCH1, NGF, GPCR) and an up-regulation of genes controlling metabolic pathways (heme metabolism, oxidative phosphorylation, protein metabolism including amino acid and derivatives, fatty acid metabolism, TCA cycle and respiratory electron transport chain). This relationship is reversed in post-operative samples comparing gene profiles in patients who develop LCOS vs those who do not. Specifically, there was an up-regulation in genes controlling inflammatory pathways and a downregulation of genes controlling metabolic pathways (Fig. 3, Supplemental Table 3).

In the GSEA results for the No-LCOS group, pre-operative and post-operative comparison revealed no changes in inflammatory gene regulation, whereas up-regulation of genes controlling metabolic pathways (TCA cycle, metabolism of proteins, mitochondrial protein import) was observed. On the contrary, comparison of pre-operative and post-operative samples for the patients who developed LCOS revealed an up-regulation of genes controlling inflammatory pathways and a downregulation of genes controlling metabolic pathways (Fig. 3, Supplemental Table 3).

### Real time PCR validation of data

**Figure 4:**
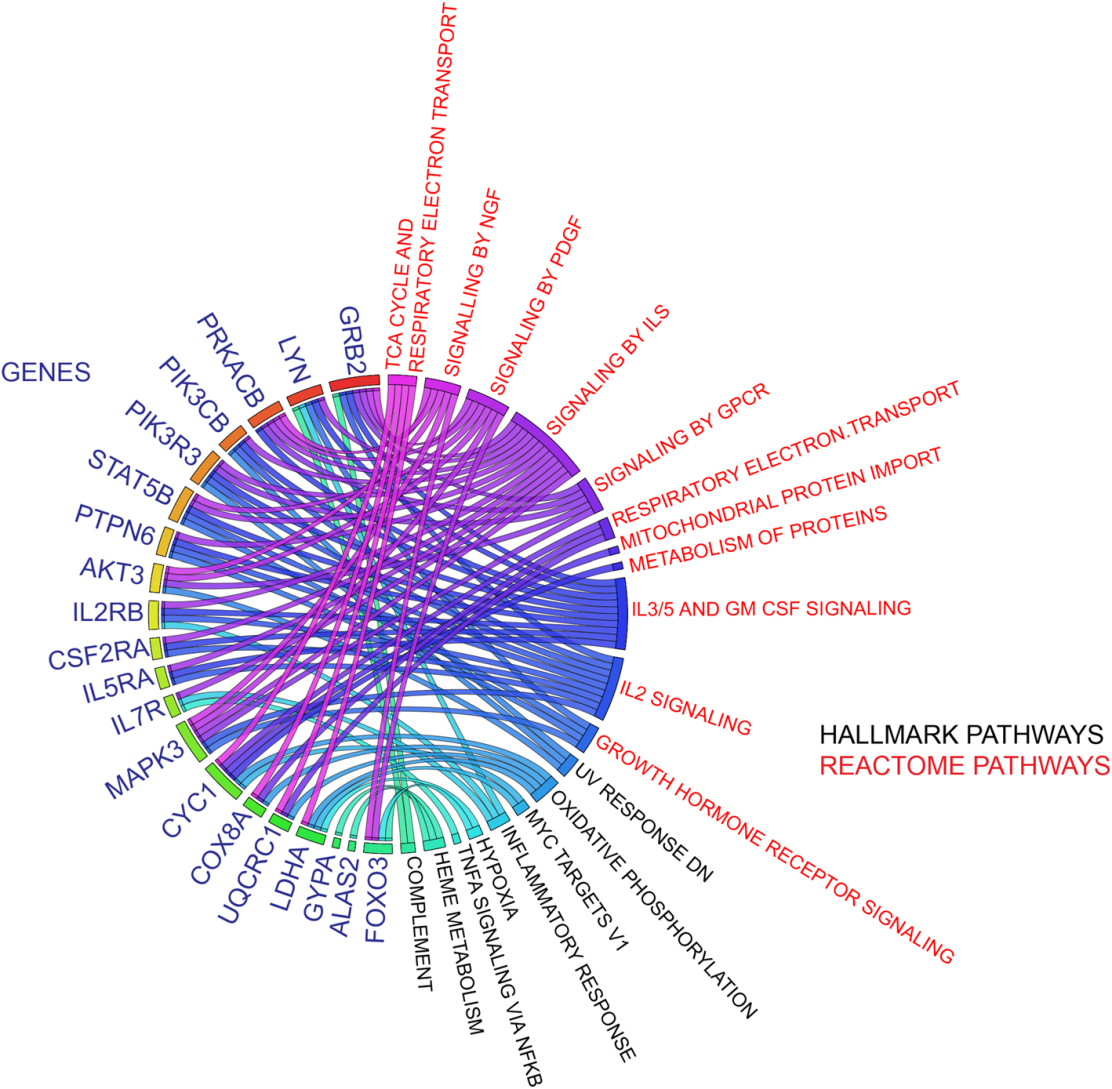
Circos plot linking the validation target genes to their associated pathways of interest. (Circos software version 0.69 and Circos Table Viewer software version 0.63, http://www.circos.ca/software/).

**Figure 5:**
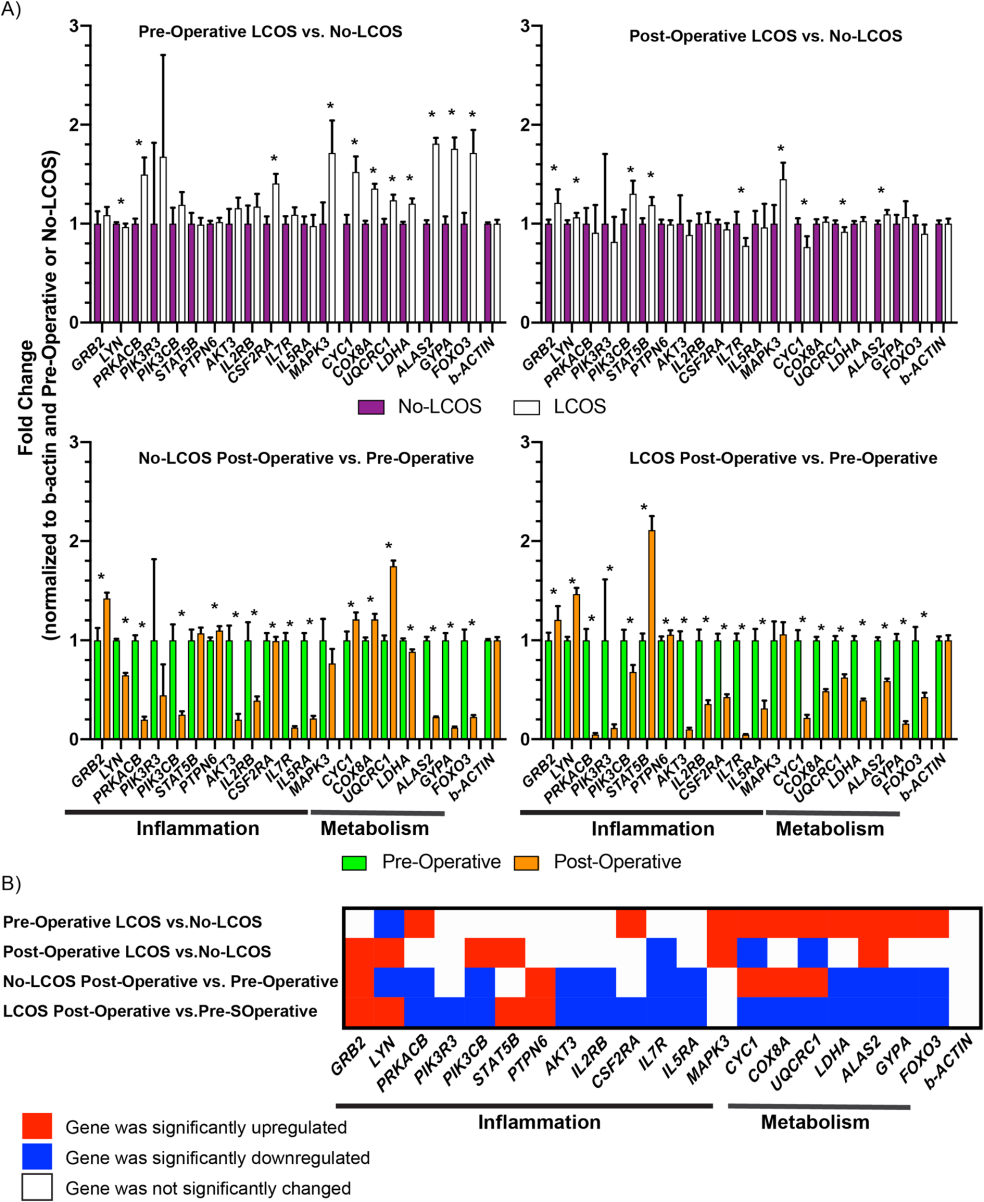
(**A**) Detailed qPCR analysis of validation genes as identified by HALLMARK and REACTOME. (**B**) Summary of the results from the qPCR validation indicating statistical significance (*p*-value < 0.05) and direction (either up or down) for the given comparison. Red indicates genes that were significantly upregulated, blue indicates genes that were significantly downregulated and white indicates genes that were unchanged.

We selected 20 genes for qPCR validation of the GSEA analysis (Supplemental Table 4). These genes were determined to be statistically significant in our differential gene analysis and they are members of the enriched inflammation and metabolic pathways as reported above. A detailed link between validation genes and differentially enriched pathways is indicated via a circos plot in Fig. 4. We emphasized two gene groups based on their corresponding pathways: inflammatory genes and metabolic genes. When comparing the pre-operative samples between the LCOS and No-LCOS groups, there was an up-regulation of *GYPA* (1.76 fold), *CYC1* (1.52 fold), *ALAS2* (1.8 fold*)* genes representing the metabolic pathway in patients with LCOS*.* On analysis of the post-operative samples between the LCOS and No-LCOS groups, this relationship was reversed with downregulation of metabolic genes such as *CYC1* (0.76 fold) and up-regulation of inflammatory pathway associated genes like *PIK3CB* (1.3 fold). (Fig. 5).

When comparing pre-operative with post-operative samples in the No-LCOS group, there was up-regulation of metabolic pathway associated genes such as *UQRC1* (1.75 fold), *COX8A* (1.21 fold), and *CYC1* (1.21 fold). In contrast, comparison of post-operative samples over pre-operative samples in patients who developed LCOS revealed down regulation of metabolic pathway associated genes like *UQRC1* (0.63 fold), *COX8A* (0.49 fold), *CYC1* (0.22 fold). Furthermore, in patients that developed LCOS there was an increase in *STAT5B* (2.11 fold) an inflammatory signaling associate gene (Fig. 5).

## Discussion

To the best of our knowledge, this is the first study that explores the whole blood transcriptome profile in neonates with HLHS undergoing the Norwood procedure. Prior studies exploring gene profile in patients with HLHS have used atrial myocardium and do not capture the evolving and dynamic clinical condition of this high risk group^31^. Whole blood transcriptomic data is potentially more relevant and useful for understanding genetic regulation of evolving clinical condition and finding likely therapeutic targets. Our data suggests that neonates with HLHS who develop post-operative LCOS have differentially regulated genes compared to those neonates who did not develop LCOS. We observed that some genes that were differentially regulated controlled the inflammatory pathway, consistent with known pathophysiological changes contributing to development of LCOS. We observed that genes controlling metabolic pathways were also differentially regulated in patients who developed LCOS compared to those without the LCOS.

A CPB-induced inflammatory response is known to be associated with post-operative morbidity, and in particular post-operative interleukins levels have served as valuable prognostic biomarkers^32,33^. Transcriptome profiles in adults after CPB have revealed increased expression of genes controlling inflammatory and complement pathways^13–15^. In our study we found increased expression of STAT5B (> twofold change), which mediates signal transduction triggered by various cell ligands, such as IL2, IL4, CSF1 and different growth hormones^34,35^. Similarly LYN and PTPN6 which control the interleukin signaling was found to be increased in patients who developed LCOS. In patients who did not develop LCOS, there was down regulation of interleukin cascades (IL7R, IL5RA, IL2RB, PRKACB) and inflammatory cascade which could explain the attenuated SIRS response. Corticosteroids have been used to attenuate the inflammatory response after cardiopulmonary bypass with mixed results in both adults and pediatric patients^36^. In a recently published randomized controlled trial using corticosteroids in neonates undergoing CPB, steroids were shown to improve outcomes in neonates undergoing palliative surgery but not corrective surgery^37^, Differential gene expression after cardiopulmonary bypass can explain individual variation in response to corticosteroids.

In our cohort we found differential regulation of metabolic pathways both at pre-operative and post-operative phases. There was dysregulation in protein metabolism including amino acid and its derivatives, fatty acid metabolism and respiratory electron transport chain. We hypothesize the altered metabolic response is the result of low cardiac output state leading to hypoperfusion compounded with anerobic metabolism that our patient population experiences due to baseline low blood oxygen saturation. Metabolomic studies in neonates suffering from hypoxemic ischemic insult have demonstrated distinct alteration in protein and fatty acid metabolism^38^. Chu et al. found that neonates with poor outcomes after hypoxemic insult have altered metabolic profile related to oxidative stress pathways and tissue damage including increased concentration of glutarate, methylmalonate, 3-hydroxy-butyrate and orotate compared to neonates with good outcome. Similarly Walsh et al. has found altered metabolic profiles involving amino acids, acylcarnitines and glycerophospholipids in neonates with poor outcomes after hypoxemic ischemic insult^39^. These metabolic profile serve as potential diagnostic and prognostic biomarkers for neonates with hypoxemic ischemic encephalopathy.

Metabolic profiling of children undergoing cardiac surgery has identified novel pathways that predict post-operative morbidity and mortality^40,41^. These studies noted shift in metabolic fingerprinting related to protein metabolism from baseline to immediately after cardiac surgery. When comparing non-survivors to survivors, Davidson et al. found differences in nicotinamide and aspartate metabolism to be associated with poor outcomes. Interestingly, tight glycemic control did not alter the metabolic and inflammatory profile in these patients^41^.

Our study found similarly distinct metabolic fingerprinting involving protein and fatty acid metabolism. Patients who developed LCOS showed statistically significant down-regulated pathways involving TCA cycle, amino acid metabolism and mitochondrial pathways. Fatty acid metabolism under aerobic condition utilizes acylcarnitine pathways, wherein they enter the electron transport chain to produce ATP. In hypoxemic conditions, fatty acid metabolism is altered energy production and accumulation of metabolites which can be directly toxic^39^. In our study there was down-regulation of CYC1 which controlled both protein metabolism and mitochondrial protein import. Down-regulation of UQCRC1, which is related to respiratory electron transport chain and ATP synthesis, along COX8A which is involved in electron transport chain play an important role in oxidative phosphorylation^42,43^. Altered myocardial function, which is primarily dependent on fatty acid metabolism, play an important role in contributing to normal cardiac output^44^. Down regulation of these genes can potentially lead to altered energy kinetics which could potentially contribute to LCOS state. These genes can potentially serve as diagnostic and prognostic biomarkers as well as therapeutic targets in children with congenital heart disease.

### Limitations

The sample size for this study is small, hence we cannot confidently generalize the data to other neonatal populations. Additionally, our cohort consistent of predominantly male patients. The effect of sex on transcriptome profile in congenital heart disease is not known. However, we developed this study as feasibility experiment. The data show that transcriptome analysis is consistent with published literature related to altered inflammatory homeostasis in children with CHD undergoing surgery with CPB. Future studies including larger numbers of patients would be needed to further validate the results of this study. The metabolic transcriptome dysregulation data was not completely validated with proteomic analysis. However, this will serve as target for our future studies exploring metabolic alteration in patients with congenital heart disease, specifically related to protein and fatty acid metabolism along with altered mitochondrial function. Finally, a potential source of the increased variability in transcriptomic response of the LCOS samples induced by surgery could be epigenomic differences between LCOS and No-LCOS groups, which is outside the scope of our current study.

## Conclusions

Patient with HLHS undergoing Norwood procedure and developing LCOS have altered transcriptome profile related to inflammatory and metabolic pathway when compared to those who did not develop LCOS. Future studies involving transcriptomic, metabolomic and proteomic data can identify novel pathways and potential therapeutic targets to improve outcomes in this high-risk population.

## Supplementary Information

Supplementary Figure.

Supplementary Tables.
