# Supplementary material for: Altered metabolic and inflammatory transcriptomics after cardiac surgery in neonates with congenital heart disease
Source: Sci Rep. 2021 Mar 2;11:4965. doi: 10.1038/s41598-021-83882-x (PMC7925649; doi:10.1038/s41598-021-83882-x)
Supplement: Supplementary file 1 — Supplementary Figure. [file 41598_2021_83882_MOESM1_ESM.docx]

**
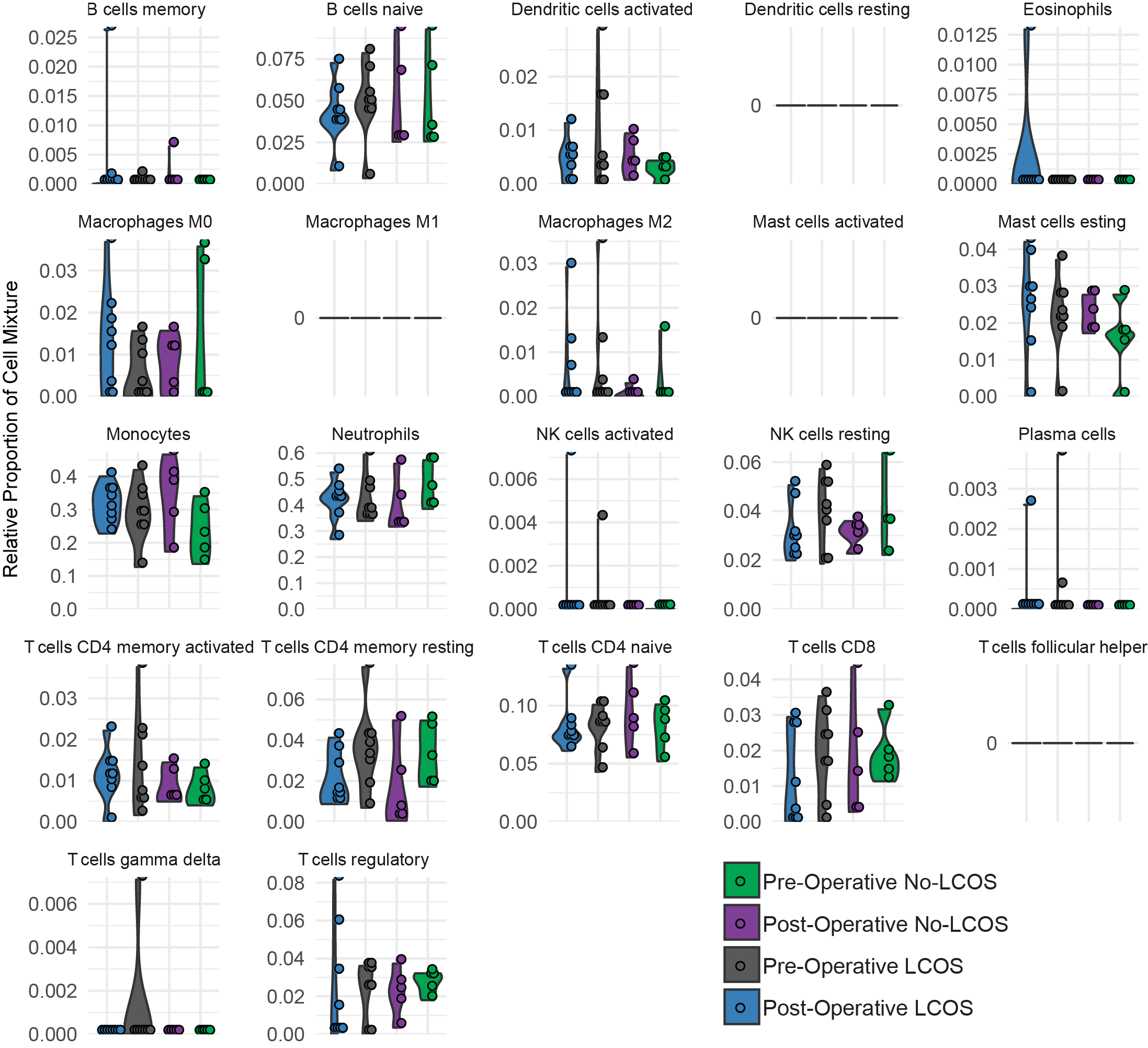
**

Supplemental Figure 1. Inferred relative abundance for 22 different immune cell types in the bulk whole blood RNA-seq results from the four distinct patient groups, showing no significant difference between the 4 groups.
